# Supplementary material for: Empirical methods for controlling false positives and estimating confidence in ChIP-Seq peaks
Source: BMC Bioinformatics. 2008 Dec 5;9:523. doi: 10.1186/1471-2105-9-523 (PMC2628906; doi:10.1186/1471-2105-9-523)
Supplement: Additional File 1 — A variety of html documents from the USeq web site detailing the available applications, their best usage, output file type descriptions, command line menus, etc. [file 1471-2105-9-523-S1.zip › USeqUserGuides/JavaDoc.zip!/JavaDoc/bioroot/oligo/OligoBean.html]

OligoBean


|  |  |  |
| --- | --- | --- |
| **Overview** \| **Package** \| **Class** \| **Use** \| **Tree** \| **Deprecated** \| **Index** \| **Help** | |  |
| **PREV CLASS**   **NEXT CLASS** | **FRAMES**    **NO FRAMES**     **All Classes** |  |
| SUMMARY: NESTED \| FIELD \| CONSTR \| METHOD | DETAIL: FIELD \| CONSTR \| METHOD |  |


---


## bioroot.oligo Class OligoBean

```
java.lang.Object
  bioroot.ReagentBean
      bioroot.oligo.OligoBean
```

**All Implemented Interfaces:**: java.lang.Comparable

---

``` public class OligoBean extends ReagentBean ```

**Author:**
:   nix
    Container for holding oligo information

---

**Field Summary**

**Fields inherited from class bioroot.ReagentBean**

`log`


**Constructor Summary**

`OligoBean()`

`OligoBean(int id,
DBUtil bioRoot)`

`OligoBean(java.sql.ResultSet results)`

`OligoBean(java.lang.String[] cells,
IdNameMatch[] genes,
IdNameMatch[] organisms,
UserBean userBean,
DBUtil bioRoot)`
  
          For bulk file upload should be 17? cells
Name, Alias Oligo Sequence, Matching Gene Sequence, uM Stock Concentration, ng/uL Stock Concentration, Volume(uL), Purification,
0 1 2 3 4 5 6 7
Modifications, Orientation, Type, Storage Location, Availibility, VisibilityBean, Handling Instructions, Target Gene Name, Organism, Notes
8 9 10 11 12 13 14 15 16 17


| **Method Summary** | |
| --- | --- |
| `double` | `getBasicTm(java.lang.String seq)` |
| `java.lang.String` | `getConcentration()` |
| `java.lang.String` | `getConcentrationUnits()` |
| `double` | `getGC(java.lang.String seq)` |
| `int` | `getGeneId()` |
| `java.lang.String` | `getGeneName(DBUtil bioRoot)` |
| `java.lang.String` | `getGeneSequence()` |
| `java.lang.String` | `getHandlingInstructions()` |
| `int` | `getLength()` |
| `java.lang.String` | `getModifications()` |
| `double` | `getMW(java.lang.String seq)` |
| `double` | `getNearestNbrTm(java.lang.String seq)`             Returns -1 if non GATC's are found. |
| `double` | `getNgUl()` |
| `java.lang.String` | `getOrientation()` |
| `java.lang.String` | `getPurification()` |
| `java.lang.String` | `getRestrictionCutSites(Enzyme[] enzymes)` |
| `java.lang.String` | `getSequence()` |
| `java.lang.String` | `getType()` |
| `double` | `getUM()` |
| `java.lang.String` | `getUMNgUl()` |
| `java.lang.String` | `getVolume()` |
| `boolean` | `isOligoNameUnique(DBUtil bioRoot)`             checks to see if oligo name is unique within labgroup, assumes a connection has been made |
| `void` | `loadOligoBean(java.sql.ResultSet results)` |
| `void` | `setConcentration(java.lang.String d)` |
| `void` | `setConcentrationUnits(java.lang.String string)` |
| `void` | `setGeneId(int i)` |
| `void` | `setGeneName(java.lang.String string)` |
| `void` | `setGeneSequence(java.lang.String string)` |
| `void` | `setHandlingInstructions(java.lang.String string)` |
| `void` | `setModifications(java.lang.String string)` |
| `void` | `setNgUl(double d)` |
| `void` | `setOrientation(java.lang.String string)` |
| `void` | `setPurification(java.lang.String string)` |
| `void` | `setSequence(java.lang.String string)` |
| `void` | `setType(java.lang.String string)` |
| `void` | `setUM(double d)` |
| `void` | `setVolume(java.lang.String d)` |
| `boolean` | `submitNew(DBUtil bioRoot)` |
| `boolean` | `updateOld(DBUtil bioRoot, UserBean submitter)` |

**Methods inherited from class bioroot.ReagentBean**

`appendMessages, canModifyAll, canModifyCommon, canView, compareTo, getAlias, getAvailability, getComments, getEditHistory, getGenericVisibility, getId, getLabGroupId, getLabGroupName, getLastUser, getLocation, getMessages, getName, getNotes, getOrderBy, getOrganismId, getOrganismName, getOrganization, getOwnerId, getOwnerName, getReference, getVisibility, isComplete, isReagentNameUnique, loadReagentBean, setAlias, setAvailability, setComments, setComplete, setEditHistory, setId, setLabGroupId, setLabGroupName, setLastUser, setLocation, setMessages, setName, setNotes, setOrderBy, setOrganismId, setOrganismName, setOwnerId, setOwnerName, setReference, setVisibility, updateCommonAccessFields`

**Methods inherited from class java.lang.Object**

`equals, getClass, hashCode, notify, notifyAll, toString, wait, wait, wait`

**Constructor Detail**

### OligoBean

```
public OligoBean()
```

---


### OligoBean

```
public OligoBean(int id,
                 DBUtil bioRoot)
```

---


### OligoBean

```
public OligoBean(java.sql.ResultSet results)
```

---


### OligoBean

```
public OligoBean(java.lang.String[] cells,
                 IdNameMatch[] genes,
                 IdNameMatch[] organisms,
                 UserBean userBean,
                 DBUtil bioRoot)
```

:   For bulk file upload should be 17? cells
    Name, Alias Oligo Sequence, Matching Gene Sequence, uM Stock Concentration, ng/uL Stock Concentration, Volume(uL), Purification,
    0 1 2 3 4 5 6 7
    Modifications, Orientation, Type, Storage Location, Availibility, VisibilityBean, Handling Instructions, Target Gene Name, Organism, Notes
    8 9 10 11 12 13 14 15 16 17


**Method Detail**

### getMW

```
public double getMW(java.lang.String seq)
```

---


### getGC

```
public double getGC(java.lang.String seq)
```

---


### getLength

```
public int getLength()
```

---


### getRestrictionCutSites

```
public java.lang.String getRestrictionCutSites(Enzyme[] enzymes)
```

---


### getBasicTm

```
public double getBasicTm(java.lang.String seq)
```

---


### getNearestNbrTm

```
public double getNearestNbrTm(java.lang.String seq)
```

:   Returns -1 if non GATC's are found.

---


### getGeneName

```
public java.lang.String getGeneName(DBUtil bioRoot)
```

---


### getGeneId

```
public int getGeneId()
```

---


### getUMNgUl

```
public java.lang.String getUMNgUl()
```

---


### getConcentrationUnits

```
public java.lang.String getConcentrationUnits()
```

---


### setConcentrationUnits

```
public void setConcentrationUnits(java.lang.String string)
```

---


### getConcentration

```
public java.lang.String getConcentration()
```

---


### setConcentration

```
public void setConcentration(java.lang.String d)
```

---


### getUM

```
public double getUM()
```

---


### setNgUl

```
public void setNgUl(double d)
```

---


### getNgUl

```
public double getNgUl()
```

---


### setUM

```
public void setUM(double d)
```

---


### loadOligoBean

```
public void loadOligoBean(java.sql.ResultSet results)
```

---


### submitNew

```
public boolean submitNew(DBUtil bioRoot)
```

---


### updateOld

```
public boolean updateOld(DBUtil bioRoot,
                         UserBean submitter)
```

---


### isOligoNameUnique

```
public boolean isOligoNameUnique(DBUtil bioRoot)
```

:   checks to see if oligo name is unique within labgroup, assumes a connection has been made

---


### getGeneSequence

```
public java.lang.String getGeneSequence()
```

---


### getHandlingInstructions

```
public java.lang.String getHandlingInstructions()
```

---


### getModifications

```
public java.lang.String getModifications()
```

---


### getOrientation

```
public java.lang.String getOrientation()
```

---


### getPurification

```
public java.lang.String getPurification()
```

---


### getSequence

```
public java.lang.String getSequence()
```

---


### getType

```
public java.lang.String getType()
```

---


### getVolume

```
public java.lang.String getVolume()
```

---


### setGeneName

```
public void setGeneName(java.lang.String string)
```

---


### setGeneSequence

```
public void setGeneSequence(java.lang.String string)
```

---


### setHandlingInstructions

```
public void setHandlingInstructions(java.lang.String string)
```

---


### setModifications

```
public void setModifications(java.lang.String string)
```

---


### setGeneId

```
public void setGeneId(int i)
```

---


### setOrientation

```
public void setOrientation(java.lang.String string)
```

---


### setPurification

```
public void setPurification(java.lang.String string)
```

---


### setSequence

```
public void setSequence(java.lang.String string)
```

---


### setType

```
public void setType(java.lang.String string)
```

---


### setVolume

```
public void setVolume(java.lang.String d)
```


---


|  |  |  |
| --- | --- | --- |
| **Overview** \| **Package** \| **Class** \| **Use** \| **Tree** \| **Deprecated** \| **Index** \| **Help** | |  |
| **PREV CLASS**   **NEXT CLASS** | **FRAMES**    **NO FRAMES**     **All Classes** |  |
| SUMMARY: NESTED \| FIELD \| CONSTR \| METHOD | DETAIL: FIELD \| CONSTR \| METHOD |  |


---
